# In Vitro Culture of Human Norovirus in the Last 20 Years

**DOI:** 10.3390/biomedicines12112442

**Published:** 2024-10-24

**Authors:** Chao Cheng, Xia Cai, Jingjing Li, Xiaomeng Zhang, Youhua Xie, Junqi Zhang

**Affiliations:** 1 MOE/NHC/CAMS Key Laboratory of Medical Molecular Virology, Shanghai Institute of Infectious Disease and Biosecurity, School of Basic Medical Sciences, Shanghai Medical College, Fudan University, Shanghai 200032, China; 23211010048@m.fudan.edu.cn (C.C.); 22211010049@m.fudan.edu.cn (J.L.); 24211010055@m.fudan.edu.cn (X.Z.); 2 Biosafety Level 3 Laboratory, Shanghai Medical College, Fudan University, Shanghai 200032, China; xiacai@fudan.edu.cn

**Keywords:** HuNoVs, in vitro culture, enteroid, organoid

## Abstract

Human noroviruses (HuNoVs) are the main pathogens that cause acute gastroenteritis and lead to huge economic losses annually. Due to the lack of suitable culture systems, the pathogenesis of HuNoVs and the development of vaccines and drugs have progressed slowly. Although researchers have employed various methods to culture HuNoVs in vitro in the last century, problems relating to the irreducibility, low viral titer, and non-infectiousness of the progeny virus should not be ignored. In 2016, researchers achieved the cultivation and successive passaging of some HuNoV genotypes using human intestinal enteroids, initially demonstrating the potential use of organoids in overcoming this challenge. This paper reviews the efforts made in the last 20 years to culture HuNoVs in vitro and discusses the superiority and limitations of employing human intestinal enteroids/organoids as an in vitro culture model for HuNoVs.

## 1. Introduction

**Figure 1:**
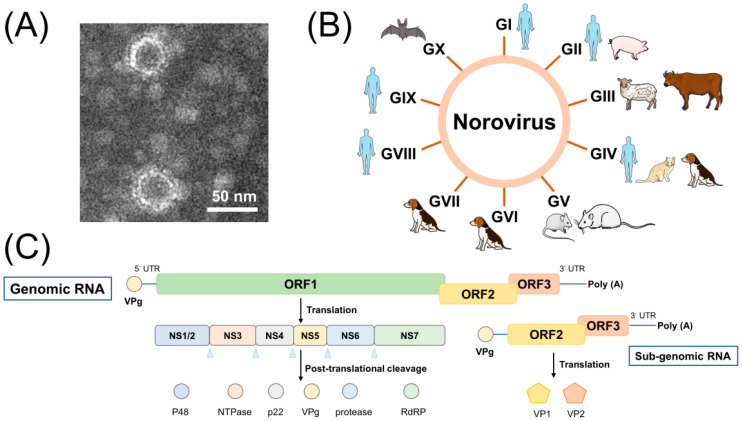
Morphological characteristics, host range and genomic features of noroviruses: (**A**) morphology of HuNoV GII.4 VLP under transmission electron microscopy, showing an ortho icosahedral structure with a diameter of approximately 40 nm; (**B**) host range of noroviruses; (**C**) genomic organization of HuNoVs. The RNA genome of HuNoVs can be divided into three ORFs that encode the precursors of six non-structural proteins, the major structural protein VP1 and the minor structural protein VP2.

Noroviruses (NoVs) belong to the genus Norovirus of the family *Caliciviridae*. The viral particles have an icosahedral symmetric structure with T = 3 and a diameter of approximately 25–40 nm and consist of 90 VP1 capsid protein dimers and a small number of VP2 complexes without an envelope (Figure 1A) [1,2]. The viral genome is positive-sense single-stranded RNA of approximately 7.7–8.3 kb, which binds to the virally encoded protein VPg at the 5′ end and is polyadenylated at the 3′ end [3,4,5]. Based on genetic characterization, NoVs can be classified into 10 genogroups and 49 genotypes [6], and there are five strains associated with human infections (known as human noroviruses (HuNoVs)): GI, GII, GIV, GVIII and GIX (Figure 1B) [6,7,8,9]. GII.4 is characterized by a rapid rate of mutation and is associated with 50% of global norovirus outbreaks [10], with this genotypic strain causing six pandemics since the 1990s [11,12]. During the winter of 2014, the GII.17 strain, which escaped population immunity, emerged in Asia, causing widespread outbreaks in China and Japan and replacing GII.4 in some areas [13,14]. In recent years, non-GII.4 viruses such as GII.2 and GII.3 have also been observed to cause large outbreaks in several countries [15].

HuNoVs mainly transmit through the fecal–oral route, and are characterized by a high transmission capacity (the minimum infectious dose is less than 100 viral particles) [16], a wide range (can be transmitted through human sources, food sources, water sources, etc.) [17], and a long survival time (can survive on the surface of the object for 2 weeks and in the environment for 9 weeks) [18]. It has been estimated that HuNoVs cause nearly 700 million illnesses and more than 200,000 deaths per year worldwide [19,20,21]. HuNoVs infect people of all ages, and infected individuals typically experience nausea, vomiting with watery non-bloody diarrhea, abdominal cramps, and fever and malaise within 12–24 h [22,23,24,25]; in these cases, HuNoVs are excreted in large quantities in feces (10^5^–10^11^ virus copies per gram of feces) and vomit (10^3^–10^6^ virus copies per milliliter of vomit) [26,27,28,29]. Although immunocompetent people usually recover from infection within 2–3 days [30], recurrent infections and the increasing prevalence of new strains are detrimental to health. For the elderly and young children, infection with HuNoVs can have serious consequences, such as gastroenteritis and even death [31,32,33,34,35]. For immunocompromised people or solid organ transplant (SOT) recipients, chronic HuNoVs infections may occur, with viral shedding lasting weeks to years [36,37,38]. Despite the serious health problems and socio-economic burden caused by HuNoVs globally, particularly in developing countries [39], no drugs have been approved for use against HuNoVs due to an incomplete understanding of the virus’ pathogenic mechanisms. In addition, the use of four vaccine candidates against several HuNoVs has been tested in clinical trials, but these are not currently licensed [40].

The HuNoV genome (Figure 1C) consists of three open reading frames (ORFs) and untranslated regions (UTRs) at both ends [41]. ORF1 is approximately 5 kb in size and encodes an approximately 200 kilodalton (kDa) precursor polyprotein that can be hydrolyzed by the virus-encoded protease NS6 into six non-structural proteins (from N-terminal to C-terminal: p48, NTPase, p22, VPg, protease, and RNA-dependent RNA polymerase (RdRP)). ORF2 and ORF3 are translated from subgenomic RNA. ORF2 is approximately 1.8 kb in size and encodes the major capsid protein VP1. Each monomer of VP1 contains a shell (S) domain and a protruding (P) domain. The S domain is highly hydrophobic and wraps around the viral RNA to form an icosahedral shell, which is linked to the P domain by a flexible hinge of approximately 10 residues in length [2,42]. The P structural domain can be subdivided into two subdomains, P1 and P2, with the P1 subdomain consisting of an α helix and eight β-sheets connecting the S structural domain to the P2 subdomain. P2 is located in the outermost layer of VP1 and consists of six antiparallel β-sheets extending from P1. It is the target of neutralizing antibodies and contains neutralizing epitopes that interact with carbohydrates and with the hematopoietic tissue antigen; in addition, it evolves under immunoselective pressure. Meanwhile, P2 is the region that determines viral antigenicity and contains the definitive receptor-binding site for murine norovirus (MNV) and the putative receptor binding site for HuNoVs [43,44,45,46]. ORF3 is approximately 0.6 kb in size and encodes the minor structural protein VP2, which is located in the interior of the viral particle and binds to the inner surface of the viral particle through interactions with the VP1 capsid structural domain; it is also thought to be involved in capsid aggregation [1,40,47,48].

**Figure 2:**
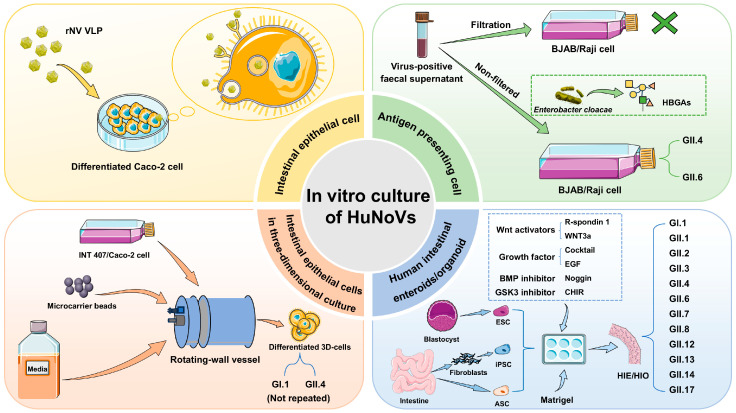
Four methods of in vitro culture for HuNoVs. Among them, intestinal epithelial cells can only achieve the attachment and internalization of HuNoV VLPs, and intestinal epithelial cells in three-dimensional culture have not been shown to be capable of culturing HuNoVs GI.1 and GII.4.

Since the discovery of HuNoVs, scientists have continuously explored in vitro culture. Although numerous studies have screened various HuNoV-sensitive cell lines, a stable in vitro replication system for HuNoVs has not been discovered [49]. At the same time, strain specificity and differences in intestinal physiology have led to a lack of suitable models showing how HuNoVs infect animals. Hence, it is difficult to address the mechanisms of HuNoV infection, their interaction with the host, the screening of antiviral drugs and the development of vaccines. In this paper, we review the four main in vitro culture systems of HuNoVs (Figure 2), and focus on the effectiveness of enteroid/organoid culture technology.

## 2. In Vitro Culture of HuNoVs

### 2.1. Culture of HuNoVs in Intestinal Epithelial Cell Lines

Intestinal epithelial cells are the first type of cells that HuNoVs encounter during their invasion from the mucosal surface to the interior. Pathological features such as shortened villi, mucosal inflammation, and the appearance of the vacuolization of intestinal epithelial cells with shortened microvilli have been observed in biopsies of the proximal small intestine of positive patients [50,51]. In addition, biopsy tissues from different parts of the intestine of a pediatric intestinal transplant patient with acute HuNoVGII.4 Sydney strain diarrhea were analyzed, and it was found that human noroviruses target enteroendocrine cells (EECs) [52], which are specialized epithelial cells with sensory and endocrine functions [53]. These indications suggested that intestinal epithelial cells are closely related to the process of HuNoV infection, so researchers initially attempted to culture HuNoVs in intestinal epithelial cells.

The infection of insect cells with baculovirus recombinants containing norovirus VP1 cDNA can produce virus-like particles (VLPs), which have a similar morphology and antigenicity to norovirus; these particles have also been found to obtain high levels of specific serum antibodies upon the inoculation of experimental animals [54]. White et al. [55] specifically bound radiolabeled recombinant norovirus virus-like particles (rNV VLPs) to 13 different cell lines, and found that differentiated human colorectal cancer cells (Caco-2) bound the most rNV VLPs, significantly more than undifferentiated Caco-2 cells and other cells; in addition, the internalization of rNV VLPs was observed. Further studies revealed that differentiated Caco-2 cells had microvillous structures and expressed H-type 1 antigen [56], which was closely associated with HuNoV infection in the population. Marionneau et al. [57] also observed the binding and internalization of rNV VLPs after the infection of differentiated Caco-2 cells expressing H type 1 structures. Meanwhile, inoculation experiments in human gastroduodenal junction tissue sections showed that rNV VLPs preferentially bind to the apical surface of cells from the pyloric and duodenum epithelia and the villi level in the duodenum of secretor individuals; this suggests that the structure of the microvillus may increase the area of viral binding and promote viral adsorption and internalization.

Although internalization into Caco-2 cells was achieved using rNV VLPs [55,58], the level of internalization of bound rNV VLPs was low (1.4% to 6.8%). Meanwhile, when Duizer et al. [49] attempted to inoculate HuNoVs into eight human intestinal epithelial cell lines, including Caco-2, he found that none of them achieved a significant increase in gene copies or the generation of new infectious viruses.

### 2.2. Culture of HuNoVs in Antigen-Presenting Cells

Antigen-presenting cells (APCs) are a group of immune cells that take up and process antigens and present them to T cells, including macrophages (MΦ), dendritic cells (DCs) and B cells. Wobus et al. [59] discovered the first strain of MNV in 2003 and subsequently found that MNV could replicate efficiently in DCs and MΦ by orally infecting STAT1^−/−^ mice; they therefore established the first cell culture system for NoVs and demonstrated, for the first time, that NoVs are widely avirulent. Although mice cannot be infected with HuNoVs, IgG- and IgA-mediated adaptive responses can be stimulated in vivo by a low-dose injection of HuNoV VLPs [60]; therefore, it is presumed that HuNoVs also generate a cellular adaptive immune response after the infection of the host. Ponterio et al. [61] cloned and expressed the ORF2 gene of the HuNoV GII.4 strain in a recombinant baculovirus expression system, prepared rNV VLPs and tested the immune response induced by the in vitro stimulation of human peripheral blood mononuclear cells (PBMCs), and found that rNV VLPs were able to induce the activation and maturation of APCs. In addition, small intestinal sections isolated from immunocompromised transplant patients demonstrated the hospitability of HuNoVs; the expression of viral structural/non-structural proteins was found in DCs, MΦ, intraepithelial lymphocytes, T cells, and enterocytes [62]. However, when HuNoVs were cultured in vitro with DCs and MΦ derived from subpopulations of monocytes obtained from susceptible individuals, as well as CD11c + DCs derived from PBMCs, it was found that some of the DCs derived from PBMCs were able to express the viral VP1 and VPg proteins, but the viral RNA copy number did not increase in all cells; this suggests that these cells do not fulfill the conditions for the efficient replication of HuNoVs in vitro [63], and that the antigens detected previously are likely to be infected enterocytes that have been phagocytosed by immune cells.

Although attempts to culture HuNoVs in blood-derived DCs and MΦ were unsuccessful, in the meantime, researchers found that the infection of wild-type mice with MNV-1 increased the number and expression of particular B cell markers in their spleens [64]. It was therefore hypothesized that B cells might also be sensitized to norovirus, and studies on human B cells cultured with HuNoVs were initiated. In 2011, Bok’s [65] inoculation of chimpanzees with HuNoVs revealed that the serum antibody response and fecal virus duration were similar to those found in humans, and the viral capsid protein was found in duodenal B cells; this suggested that B cells may be susceptible to HuNoVs. In 2014, Jones et al. [66] successfully inoculated the HuNoVs GII.4 Sydney strain, MNV-1 and MNV-3 into Burkitt lymphoma B cell lines (BJAB cell line) and achieved an increase in the viral copy number and the production of structural/non-structural proteins, further demonstrating the production of new infectious viral granules via passaging experiments. Notably, the team highlighted the multiple factors that need to be taken into account for the experiment to be successful; the inoculum had to be an unfiltered virus-positive fecal supernatant, as the presence of intestinal microorganisms is an important cofactor in HuNoV GII.4 Sydney infection. Subsequent studies have found that *Enterobacter cloacae*, a member of the intestinal flora, expresses human histo-blood group antigens (HBGAs), which are thought to be receptors for HuNoVs in humans [67]. In addition, the type of B cells involved (which can replicate in BJAB and Raji cells, but not in Namalwa and HuNS1 cell lines) [68], their status, and their density can also affect the efficiency of infection, in addition to the source of fetal bovine serum in the culture medium. Despite the initial success of this in vitro culture system, the following problems remain. Fewer HuNoV strains can be cultured (GII.4 and GII.6); the replication efficiency of the viruses is low (0.1–0.5 Log10), and is far from the level of viral replication in vivo; there is a wide variation in the replication efficiency replicated in different laboratories; and the replication efficiency of the viruses decreases as the viral starting inoculum increases [67,68,69,70]. Hence, this culture system can only moderately increase the number of HuNoV genome copies and is unlikely to produce the levels of viral replication observed in vivo [66].

HuNoVs can infect immunodeficient patients and achieve efficient replication in the intestinal epithelium, while CD20+ B cells have not been detected in tissue sections [62]. The viral titer was found to be approximately one log lower in patients lacking B cells compared to those with B cells [71]. Studies in mice have found that VP2 of HuNoVs specifically antagonizes the presentation of antigens by B cells to CD8+ T cells, which are essential in controlling acute HuNoV infection [72]. Furthermore, human B cells infected with HuNoVs do not lose cell viability, which contrasts with the lysis of macrophages and dendritic cells that occurs after infection with MNV [59,66,67]. In summary, B cells play an important role in HuNoV infection and contribute to the viral load. Presumably transcytosis occurs as the virus passes through the intestinal epithelium and enters innate or adaptive immune cells [73]. Meanwhile, the replication of HuNoVs in vivo may involve a variety of cells and take place in a specific environment, which is why it is difficult to employ single, two-dimensional (2-D) cell lines to achieve the efficient culture of HuNoVs in vitro.

### 2.3. Culture of HuNoVs in 3D Cell Aggregates

For decades, 2D monolayer cell culture systems for HuNoVs have ended in failure or inefficiency, suggesting that conventional cultures cannot be used for effective infection and replication. In living animals infected with the virus, cells function together in a three-dimensional (3D) environment, and this spatial environment may be critical for the infection of viruses, or even other infectious pathogens [74,75,76]. In addition, conventional 2D monolayers lack the polarity and certain structural features of in vivo tissue [77,78], such as tight junctions, mucus, microvilli and mucins, which have been shown to play an important role in viral infection, replication and delivery processes [79,80,81,82]; this makes it challenging to accurately and comprehensively model virus–host interactions.

In 1998, the National Aeronautics and Space Agency (NASA) reported the use of a rotating wall vessel (RWV) bioreactor [83] that simulates a microgravity, low-fluid shear environment, allowing cells cultured within it to form polarized and 3D aggregate forms; this enabled the structure of native tissues to be effectively recreated and the human microenvironment to be mimicked [75,84]. Since its introduction, researchers have used the reactor to culture aggregates of cells from numerous organs (lymphatic, lung, kidney, gastric, intestinal, vaginal, etc.) and to delve deeper into the pathogenesis of the viruses involved. In particular, when two aggregates of 3D monkey renal epithelial cells (VERO) and 3D bovine renal epithelial cells (MDBK) were infected with two types of RNA virus (vesicular stomatitis virus and bovine parainfluenza virus) and two types of DNA virus (herpesvirus 1 and bovine adenovirus), it was found that the aggregates of cells were more resistant to viruses than monolayers of cells; in addition, once the aggregates were successfully infected, a more infectious virus would be produced [74]. This was further confirmed in other cell aggregates [85,86,87,88,89], demonstrating the superiority of using 3D aggregates to model real in vivo environments.

In order to create an infection environment closer to that of living organisms, Straub et al. [90] applied the RWV technique and successfully cultured two strains (GI.1 and GII.4) of HuNoVs in 3D aggregates of human embryonic small intestinal epithelium (INT 407) with multiple passages. One day after being infected with HuNoVs, the cell aggregates exhibited vacuolization and shortened microvilli, detaching from cytodex beads; they also showed cell pathological effects (CPEs), with the cell morphology becoming elongated or distorted. Virus particles mainly accumulate in tissue aggregates. Subsequently, the team cultured two strains of HuNoVs, GI.1 and GII.4, in 3D aggregates of Caco-2; these showed 2–3 Log10 viral RNA copy numbers in the infected aggregates and the infected cells were localized via immunoelectron microscopy [91]. However, none of the other laboratories supported the indication of viral replication after culturing 3D aggregates of INT 407 or Caco-2 cells using the same method; however, cell differentiation and the formation of apical villi were observed [92,93]. Herbst et al. [94] did not observe viral VP1 and VPg using confocal microscopy, and immunofluorescence did not detect HBGAs in INT 407 cells. This demonstrates that the previously observed CPEs were most likely caused by the contamination of the samples with cytotoxic molecules, such as lipopolysaccharide (LPS) and bacterial endotoxin, rather than viral infection.

### 2.4. Culture of HuNoVs in HIE/HIOs

Enteroids and organoids refer to the growth and differentiation of adult stem cells (ASCs), embryonic stem cells (ESCs) or induced pluripotent stem cells (iPSCs) into organs with histological and genetic characteristics by adding various factors essential for stem cell maintenance (R-spondin 1, Noggin, epidermal growth factor (EGF), activators, inhibitors, etc.) to the culture medium in a simulated 3D environment in vitro. The technique was pioneered by Hans Clevers’ team [95] in 2009, when mouse Lgr51+ intestinal stem cells were successfully cultured in a non-epithelial ecological niche in vitro into villous intestinal epithelial structural domains with multiple differentiated cell types (stem cells, Paneth cells, enterocytes, goblet cells and enteroendocrine cells), which have the ability to grow over long periods of time. This technology has evolved rapidly since then, with the creation of intestines [96], brains [97], stomachs [98], lungs [99], ovaries [100], and many other types of organoids. Because organoids can simulate and reflect the state of human organs in different environments, they are used in many fields such as pathogenic microbial infections, drug development and screening, regenerative medicine and precision medicine.

Human intestinal enteroids (HIEs) consist of mesenchyme and epithelial layers, which contain the major cell types of the intestinal epithelium with crypt-like proliferative zones. Combining the high throughput of epithelial monolayer cell lines with the complexity of the gut in experimental animals, HIEs have been widely used in studies of human gut-associated diseases, and provide a new direction for the in vitro culture of various viruses [101].

In 2012, Mary K. Estes’ team obtained induced human intestinal organoids (HIOs) via the directed differentiation of ESCs and successfully inoculated rotavirus to produce infectious progeny viruses. This was the first report of microbial cultures using organoids [102]. The experiment also revealed that rotavirus could infect mesenchymal cell populations, demonstrating the applicability and unique superiority of organoids as a viral culture system. Four years later, the team cultured monolayer HIEs of jejunal origin, and the system effectively supported the replication of different subtypes of HuNoVs (after 96 h, the HuNoV genomic RNA increased by 1.5–2.5 log10, with 35–45% of the cells infected, and a typical viral particle structure under transmission electron microscopy) and passaging (four consecutive passages showing the birth of infectious progeny of the virus). The researchers found that additives such as bile acid enabled the replication of both subtypes GII.3 and GII.17 and promoted the replication of subtype GII.4; meanwhile, heat or radiation treatments effectively inactivated HuNoVs [103]. This study represented a major breakthrough in the in vitro culture of HuNoVs, greatly advancing basic research on HuNoVs. The experimental results have been successfully validated by multiple laboratories in different countries, successfully supporting the replication of multiple genotypes of HuNoVs (GI.1, GII.1, GII.2, GII.3, GII.4, GII.6, GII.8, GII.12, GII.14 and GII.17) [104,105,106,107,108]. The replication of HuNoVs in HIEs is biologically relevant, as the replication of the GII.4 strain is restricted to cultures from secretor individuals, whereas the GII.3 strain replicates from both secretor and non-secretor individuals; this is in line with the results of epidemiological studies [27,109]. In addition, undifferentiated HIE cultures predominantly contain stem cells, but HuNoVs do not replicate in these cells, consistent with the lack of HuNoVs detected in stem cells in the crypts of infected humans or animals [62,110,111]. Unlike primary tissue culture, HIEs can be maintained using serial passaging and stored for later use [112]. Human jejunal intestinal enteroids isolated and obtained in 2012 were still available for experimental use after 7 years, indicating that HIE cultures are extremely stable [106]. More importantly, the replication of HuNoVs in HIEs reflects epidemiological differences in host susceptibility that depend on genetic variations in the expression of HBGAs associated with the donor’s secretory status [113]. However, the disadvantages of this culture method include the time-consuming construction of enteroids, the high cost of maintaining the culture, the high number of HuNoVs that need to be inoculated (with a minimum of 1 × 10^5^ genome equivalents), and the low titer of the HuNoVs produced [105]. In addition, access to living human tissues is not easy for many laboratories and it has ethical implications, making it necessary to optimize this culture system.

In 2019, Sato et al. [114] used human iPSCs to construct monolayered, polarized primary intestinal epithelial organoids and successfully inoculated them with HuNoVs (GII.3, GII.4, GII.6, and GII.17) and VLPs. They found that the replication of HuNoV GII.3 and GII.17 in the HIO was not bile-dependent, suggesting that biological differences in organoids and enteroids (iPSCs or ASCs) can lead to different responses to external stimuli, the mechanisms of which are worth further investigation. Mirabelli et al. [115] constructed 2D and 3D HIEs using fetal jejunal tissues and inoculated them with HuNoV GII.4, finding that the viral yields of the two culture systems were 1 log10 and 2.5 log10, respectively. Compared with the 2D system, which requires the microinoculation of the virus, 3D HIEs can spontaneously undergo polarity reversal and directly infect the virus (no microinjection is required). In addition, the pre-application of different drugs to 3D HIEs prior to the inoculation of the virus can promote (ruxolitinib, a JAK1/2-inhibitor) or inhibit (polymerase inhibitor 2′-C-Methylcytidine) the replication of the virus significantly, suggesting that 3D HIEs are a promising platform for the testing of anti-HuNoV drugs.

**Figure 3:**
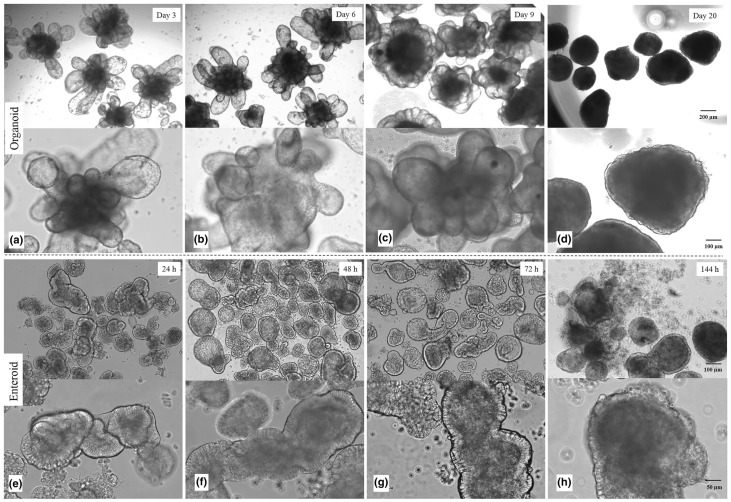
The morphology of organoids and enteroids at different developmental phases [116]. The morphology of developing organoids. Finger-like projections of organoids on day 3 (**a**), shorter villus-like organoids on day 6 (**b**), round budding organoids with tightly arranged crypt cells on day 9 (**c**), spherical organoids on day 20 (**d**); Enteroids with clear and compact crypt cells at 24 h (**e**), round enteroids with a cavity at 48 h (**f**), increasing scattered cells from enteroids at 72 h (**g**), numerous repelled cells and atypical crypt cells at 144 h (**h**).

Figure 3 shows the natural morphology of fetal bovine intestinal organoids and enteroids at different developmental phases [116]. The morphological features of both the organoids and enteroids are dynamic, and both of them have a distinct morphology. Table 1 compares the origin, characteristics, and current cultured HuNoV genotypes of HIEs and HIOs. Although HIOs are more readily available than HIEs, which can only be obtained via the generation of biopsy tissue, their inclusion of mesenchymal elements and the fetal phenotype may have implications for their use [117]; in addition, unlike HIEs, HIOs cannot be preserved over an unlimited number of generations [112,118,119]. In conclusion, HIOs and HIEs have greatly contributed to our understanding of the mechanisms implicated in HuNoV infection [120,121,122,123,124,125]. Meanwhile, the anti-HuNoV effects of several common antiviral small-molecule compounds have been studied using HIEs (e.g., the EC50 of Dasabuvir was 7.55–9.85 µM for GII.3 and 11.71–12.41 µM for GII.4, and the EC50 of Nitazoxanide was 27.4 µM, 14.4 µM, 12.9–53.9 µM, and 33.4 µM for GI.1, GII.3, GII.4 and GII.17, respectively) [115,126,127,128,129]. However, to date, these HuNoV culture systems have struggled to achieve high viral yields (30–1000-fold increases in the viral titer) with successive passages of infection (supporting viral replication out to the fourth passage), and the HIE/HIOs infected with HuNoVs have not shown significant CPEs.

### 2.5. Superiority and Prospects of Enteroid/Organoid Culture Systems

Table 2 shows the details of several in vitro culture systems that have been reported to culture HuNoVs. Among them, the HIO/HIE culture shows advantages in terms of reproducibility and efficiency. Prior to the development of organoid technology, studies on pathogenic microorganisms were mainly based on two culture systems: specific cell lines and animal models. Although cell culture is simple and inexpensive, the 2D cell layer does not truly reflect the 3D spatial environment in which pathogenic microorganisms invade the organism, and lacks the interactions and antiviral responses of the various cell types in a tissue or organ [133,134,135]. In addition, cell lines are usually derived from tumor tissue or undergo specific mutations, thus proliferating indefinitely and not accurately representing healthy tissue [118]. Although animal models can reflect the real infection of pathogenic microorganisms in organisms, this system requires the investment of extensive time and resources; this is especially the case for special experimental animals such as green monkeys and baboons, which are difficult to evaluate in large numbers in most laboratories. When used to study microorganisms that infect humans, there are still non-negligible differences between the internal environments of these animals and the real human body, thus limiting the in vitro cultivation of some pathogenic microorganisms and the study of pathogenesis [136,137,138]. The organoid model is between cell lines and animal models, and can be cultured by ASCs, pluripotent stem cells (PSCs) or iPSCs in vitro on a large scale to obtain a multi-type cell group with an ecological niche that maintains certain characteristics and functions; it is also able to recapitulate part of the physiological functions and facilitate stable self-renewal. Compared with single cell lines, the structural characteristics of organoids can facilitate interactions and cell–matrix connections between cell types, thus creating different areas of nutrient supply and oxygen concentration gradients. Numerous studies have found that multiple viruses (echovirus 71, astrovirus) induce HIE antiviral pathways or specific interferon (IFN)-stimulated genes (ISGs) that are not found in conventional single-cell lines, suggesting that cell lines used in the laboratory for long periods of time may not fully recapitulate the physiological processes involved in viral infection [139,140]. Compared to animal models, human-derived organoids can more realistically reflect the internal environment and physiological processes after infection and can shorten the culture cycle and enable dynamic tracking. Therefore, multiple organoid models have been employed in the study of viral infectious diseases, including hepatitis B virus [141], coxsackievirus [139], and Zika virus [142]; these models have also been combined with single-cell RNA sequencing (scRNA-seq), CRISPR/Cas9, proteomics, transcriptomics and chip technology [143,144,145,146] to analyze the viral pathogenicity mechanisms, host receptors and screening used for antiviral compounds.

Meanwhile, because of the relatively short development time, organoid technology has many unsolved problems that need to be further explored and resolved. First, the current method used to prepare organoids relies on a variety of growth factors, but these are not sufficient to reproduce the body’s fine and complex microenvironment during the development of real organs. The limited concentration of oxygen and increased quantity of metabolic waste generated during the cultivation process may lead to local necrosis of organoids; the diameter of the fully grown organoids is generally 100–500 μm and they lack the vascular structure and the immune components required to generalize complex human disease models. In recent years, Bouffi et al. [147] established an organoid model that uses functional human immune tissues in humanized immune system mice, providing a new approach to the study of immunized organoids. In addition, to increase the complexity of the HIE model and more closely replicate in vivo conditions, HIE cells can be co-cultured with mesenchymal or vascular cells and gut microbiota fractions [106]. Secondly, the Matrigel protein mixture used as the culture medium was produced by BD Biosciences; this medium is expensive and the main ingredient comprises a mixture of glue-mounted proteins secreted by murine sarcoma cells. Therefore, it is worth analyzing whether this exogenous ingredient has an impact on the organoid mimicry of the real organ and whether it can be used as a reliable therapeutic means of clinical evaluation. Thirdly, although the main growth factors used were Wnt3a, R-Spondin and Noggin, the composition and proportions of the media produced by different manufacturers for the culture of HIEs are confidential; this is important for the HIE differentiation and HuNoV cultivation. Ettayebi et al. [103] compared the use of the previously described primary proliferation medium (BCMp) and differentiation medium (BCMd) with IntestiCult™, a commercially available human organoid growth medium produced by Stem Cell Technologies. They found that the HuNoVs strain in IntestiCult™ had a significantly higher replication rate [113]. Fourthly, due to the low level of automation in the organoid culture process, the likelihood of human-induced systematic error is high, and industry standards that regulate the maturation morphology and size of different types of organoids have not yet emerged. This has indirectly led to difficulties in performing organoid experiments that are reproducible and consistent. According to a study conducted in the United States, the cost of reagents for testing a single HuNoV sample in HIE culture is approximately USD 36, and the process takes at least three weeks from start to quantification [148]. However, the exploration of organoids is still ongoing, and increasingly sophisticated culture systems will lead to a more prominent role for organoids in effective in vitro models to mimic real organ development and infection.

## 3. Conclusions

HuNoVs exert an economic burden and cause health problems every year, so improving existing in vitro culture models is necessary in order to better understand, prevent and treat the viral infection. While B cells and HIE/HIO culture systems have aided in the development of vaccines and drugs, limited viral replication still restricts further research on HuNoVs. One of the most effective ways to establish in vitro cultures of HuNoVs is to identify the cellular receptor for HuNoVs and then establish cell lines overexpressing the receptor. However, it has unfortunately been proven that CD300lf, the primary physiological receptor for MNV, is not a receptor for HuNoVs [149]. Regarding the culture of HuNoVs with HIE/HIOs, the efficiency can be improved by optimizing the culture system via methods such as inducing the knockdown of STAT1 [124], specific inhibitors [122] or the overexpression of specific viral proteins [115,122]. This would inhibit the JAK-STAT pathway, one of the major players in host innate immunity. At the same time, the study of HuNoVs, alongside other disciplines such as immunology and emerging technologies such as RNAseq, will enhance our understanding of them, providing an opportunity to prevent and treat HuNoVs in the future.

## Figures and Tables

**Table 1:** Comparison of HIOs and HIEs in terms of the source, characterization, and cultivation of HuNoVs.

	HIOs	HIEs	References
Mesenchymal niche	Yes	No	[117]
Source	ESCs/iPSCs	ASCs	[96,117,130]
Time required to establish	Over a month	A few days	[131]
Term of use	Limited	Indefinite	[112,118,119]
Culturable HuNoVs	GII.3, GII.4, GII.6, GII.17	GI.1, GII.1, GII.2, GII.3, GII.4, GII.6, GII.7, GII.8, GII.12, GII.13, GII.14, GII.17	[113,114,132]

**Table 2:** Summary of the described HuNoV cultivation systems.

Culture Model	Culture Conditions	Culturable Genotypes	Viral Replication Efficiency	Repeatability	References
BJAB cell line	Unfiltered virus- positive fecal supernatant	GII.4, GII.6	10–50-fold increase	Yes	[66,67,68,69,70]
3D INT 407/Caco-2 aggregates	RWV bioreactor	GI.1, GII.4	/	No	[90,91,92,93,94]
HIEs	Bile acid is necessary for GII.3 and GII.17	GI.1, GII.1, GII.2, GII.3, GII.4, GII.6, GII.7, GII.8, GII.12, GII.13, GII.14, GII.17	30–1000-fold increase	Yes	[103,104,105,106,107,108]
HIOs	/	GII.3, GII.4, GII.6, GII.17	10–1000-fold increase	Yes	[114,132]
